# Morpho-Anatomy, In Vitro Culture, and Phylogenetic Studies of Two *Helicotylenchus* Species from Southern Alberta, Canada

**DOI:** 10.3390/microorganisms12030497

**Published:** 2024-02-29

**Authors:** Maria Munawar, Michele Konschuh, Pablo Castillo, Dmytro P. Yevtushenko

**Affiliations:** 1 Department of Biological Sciences, University of Lethbridge, 4401 University Drive W, Lethbridge, AB T1K 3M4, Canada; maria.munawar@uleth.ca (M.M.); m.konschuh@uleth.ca (M.K.); 2 Institute for Sustainable Agriculture (IAS), Spanish National Research Council (CSIC), Campus de Excelencia Internacional Agrolimentario (ceiA3), Avenida. Menendez Pidal s/n, 14004 Cordoba, Spain; p.castillo@csic.es

**Keywords:** bionomics, carrot disk, *H. crassatus*, *H. oscephalus*, morphology, morphotype, molecular characterization, SEM, spiral nematodes

## Abstract

Spiral nematodes (*Helicotylenchus* spp.) are polyphagous parasitic species exhibiting a broad host range and geographical distribution. However, their diversity in the cultivated regions of southern Alberta remains understudied. Hence, we conducted a comprehensive survey of the region’s arable lands for the presence of spiral nematodes and revealed two *Helicotylenchus* species, *H. crassatus* and *H. oscephalus*. *H. crassatus* consisted of two distinct morphotypes: one morphotype had a conoid tail with slight ventral projection on the distal end, whereas the other had a broadly rounded tail. This study presents the first documentation of *H. crassatus* and *H. oscephalus* from southern Alberta, Canada. Molecular characterization was based on the partial 18S rRNA, the D2–D3 of 28S rRNA, ITS rRNA, and *COI* gene sequences, complemented by detailed morphological studies using scanning electron microscopy. In this work, *Helicotylenchus* species were often co-detected with root lesion nematodes, which made the evaluation of their role in crop damage more difficult. To meet the requirements for threshold and pathogenicity assessments, we introduced both spiral nematode species to sterile carrot disks and evaluated the feasibility of their multiplication and mass production in vitro. The present findings expand the taxonomic records of *Helicotylenchus* spp. and improve diagnostics of these morphologically similar species. Furthermore, our in vitro culture technique will provide a reliable source of the initial inoculum for future plant–nematode interaction studies.

## 1. Introduction

The genus *Helicotylenchus* Steiner [1], commonly known as spiral nematodes, is recognized for its distinctive body appearance and stands as the largest genus within the family Hoplolaimidae [2]. The species in the genus are polyphagous, usually inhabit the rhizosphere of plants and feed as obligate ecto- or semi-endoparasites [3]. The genus exhibits amphimixis and parthenogenetic modes of reproduction, and in favorable environmental conditions, these nematodes can complete their life cycle within 32 to 39 days [4]. *Helicotylenchus* species have a cosmopolitan distribution with over 200 nominal taxa [5]. These species were documented from diverse agro-environments; however, none of the *Helicotylenchus* species were included in the regulated pest list, indicating they are not aggressive pests of plants [6]. Over the years, *H. oleae* Inserra, Vovlas and Golden [3], *H. multicinctus* (Cobb) Golden [7,8], *H. pseudorobustus* (Steiner) Golden [8,9], and *H. varicaudatus* Yuen [10], have gained scientific interest due to their potential effects on several crops [11,12]. However, robust evidence establishing their direct or indirect implications in terms of plant growth suppression and damage remains scarce in the scientific literature [6,13].

Taxonomic investigations on spiral nematodes were frequently conducted in Canada during the 1970s and early 1980s, the studies resulted in a comprehensive documentation of 17 species distributed across various regions of the country [14,15,16,17]. Among these, seven species were specifically recorded in Alberta, indicating a diverse presence of spiral nematodes in our local soil ecosystems [14]. However, it is notable that there has been a scarcity of official reports regarding the occurrence of spiral nematodes in Alberta over the past two decades. Consequently, a comprehensive nematode inventory survey was conducted in the irrigated areas of southern Alberta to study the diversity of soil nematodes, particularly the spiral nematode group. The survey led to the discovery of several populations of spiral nematodes belonging to the genus *Helicotylenchus*.

Subsequently, these populations were subjected to detailed morphological and morphometric characterizations. An analysis of morphological and morphometric data revealed that our populations closely resemble *H. crassatus* Anderson [18], and *H. oscephalus* Anderson [16]. However, *H. crassatus* presents two morphotypes, one has a conoid tail with slight ventral projections and the other with a broadly rounded tail. The literature studies were devoid of molecular characterization and scanning electron imaging studies of our detected species. In addition, we noted, these spiral nematodes were detected with root lesion and other soil nematodes. Root lesion nematodes exhibit significant prevalence in southern Alberta [19], and their co-occurrence with these spiral nematodes may aggravate plant stress.

Consequently, we conducted tests to assess the suitability of detected spiral nematodes for in vitro cultures: the availability of such a protocol will allow for the evaluation of spiral and root lesion nematode interactions in future studies. Therefore, the objectives of the present study were (i) to provide a thorough morphological characterization of the nematodes using scanning electron microscopy (SEM), (ii) to obtain ribosomal and mitochondrial DNA sequences to conduct phylogenetic studies, and (iii) to test these populations’ ability to multiply in carrot disk cultures. The findings of this study will greatly facilitate the identification and taxonomic records of these spiral nematodes in southern Alberta, Canada. Moreover, these results may offer valuable insights for the development of an inoculum for the pathogenicity testing of spiral nematodes.

## 2. Materials and Methods

### 2.1. Nematode Isolation and Morphological Studies

To investigate the diversity of plant-parasitic nematodes (PPNs) within irrigated regions of southern Alberta, a comprehensive survey was conducted in selected fields across Bow Island, Taber, and Vauxhall. Thirty core samples were systematically collected from each field employing a Dutch auger, and were subsequently combined to form composite samples. Additionally, exploratory soil sampling was extended to the natural vegetation of the fields. The collected soil samples were then appropriately stored at 4 °C within a cold storage facility of the University of Lethbridge, Alberta, Canada, until further processing. Nematodes were extracted from the soil using a modified method of Cobb’s sieving and flotation–centrifugation technique [20]. Specifically, among the diverse soil nematodes, those exhibiting morphological characteristics of the *Helicotylenchus* genus were meticulously selected and individually mounted onto slides for detailed observation and long-term preservation. To facilitate live observations, fresh specimens of each identified species were gently transferred to a droplet of distilled water, followed by gentle heat relaxation, prior to observation under a Zeiss Axioskope 40 microscope. For morphometric analyses, the nematodes were appropriately fixed, and permanent slides were meticulously prepared following by Seinhorst [21] and De Grisse [22] techniques. High-resolution images of each individual specimen were captured using a Zeiss Axioskope 40 microscope equipped with a Zeiss Axiocam 208 camera (Carl Zeiss, Jena, Germany). Precise measurements derived from these images were executed using the ZEN 3.1 (blue edition) imaging software (Carl Zeiss).

### 2.2. Scanning Electron Microscopy (SEM)

For SEM examination, the nematodes were fixed in a mixture of 2.5% formalin and 2.5% glutaraldehyde and washed three times in a 0.1 M phosphate buffer, post-fixed in 1% osmium tetroxide, dehydrated in a series of ethanol solutions and critical-point dried with CO_2_. After being mounted on stubs, the samples were coated with gold at a 5.5 nm to 22 nm thickness and the micro-graphs were made using the 15 kV operating system of Hitachi SU5000 STEM [23].

### 2.3. DNA Extraction, PCR and Sequencing

Following microscopic examination, female specimens of each species were transferred into individual 0.2 mL PCR tubes. DNA extraction was performed on single specimens as described in Maria et al. [23], and for all gene fragments, amplification was achieved using the same specimen’s DNA. Four sets of DNA primers (Integrated DNA Technologies, Coralville, IA, USA) were employed to amplify the ribosomal (18S, 28S, and ITS ribosomal RNA (rRNA)) and mitochondrial (*COI*) segments of the genes. The partial 18S rRNA gene sequence was amplified using the 1813F/2646R primer pair [24]. Amplification of the 28S rRNA gene was done by employing the D2A/D3B primer pair [25], the ITS gene was targeted with the VrainF/VrainR primer pair [26]. The *COI* gene was amplified by the JB3F [27] and JBR5 [28] primer pair. The PCR conditions for the 18S, 28S, ITS and *COI* genes precisely adhered to previously published methodologies [24,25,26,27,28]. After the PCR amplification, the resulting products were subjected to electrophoresis in 1% agarose gels and visualized by GelRed staining (Biotium, Fremont, CA, USA). Purification of the amplified DNA fragments was accomplished using the GeneJET Gel extraction kit (Thermo Fisher Scientific Baltics UAB, Vilnius, Lithuania), following the manufacturer’s guidelines. Subsequently, the purified fragments were ligated into the pJET1.2 vector (Thermo Fisher Scientific, Mississauga, ON, Canada) and introduced into competent *Escherichia coli* DH5α cells (Thermo Fisher Scientific, Carlsbad, CA, USA). The successful insertion of PCR-derived fragments into the plasmids from transformed *E. coli* cells was confirmed via PCR analysis. The plasmid DNA was then isolated and purified using the GeneJET Plasmid Miniprep Kit (Thermo Fisher Scientific Baltics UAB, Vilnius, Lithuania) according to the manufacturer’s instructions. The isolated plasmid DNA was sent to Azenta Inc. for DNA sequencing (South Plainfield, NJ, USA). The acquired DNA sequences were aligned using a Bioedit 7.2 sequence alignment tool and compared to the known nematode species sequences available in the GenBank database for taxonomic identification and similarity assessment.

### 2.4. Phylogenetic Studies

The DNA sequences of ribosomal and nuclear genes were acquired for each detected *Helicotylenchus* species. These newly acquired sequences, and other *Helicotylenchus* species sequences accessible within the GenBank repository, were collected for subsequent phylogenetic analysis. The selection of outgroup taxa for each dataset was determined based on precedent studies [5,6]. Multiple sequence alignments were carried out by the heuristic progressive method FFT-NS-2 algorithm incorporated within MAFFT v7.450 [29]. The BioEdit v7.2.5 software [30] was employed for reviewing the sequences, the inadequately aligned positions were edited following a judicious filtering strategy (removing up to 20% of alignment positions). This filtering approach, as suggested by Tan et al. [31], is anticipated to exert minimal influence on the precision of the resulting phylogenetic trees while optimizing computational efficiency. Automated filtering methods for multiple sequence alignments are known to potentially compromise the fidelity of single-gene phylogenetic inference [31]. Phylogenetic analyses of the sequence datasets were performed using the Bayesian inference (BI) as implemented in MrBayes v3.1.2. The most fitting model of the DNA evolution for each dataset was determined using JModelTest v2.1.7 [32], employing the Akaike Information Criterion (AIC). Consequently, the selected models were as follows: (1) the general time-reversible model and a gamma-shaped distribution (GTR + G) for the D2–D3 expansion segments of 28S rRNA and ITS rRNA partial 18S, and (2) the general time-reversible model with invariable sites and a gamma-shaped distribution (GTR + I + G) for the partial 18S rRNA region and *COI* gene. These best-fit models, including the base frequencies, proportion of invariable sites, gamma distribution shape parameters, and the substitution rates derived from the AIC, were subsequently applied within MrBayes for conducting the phylogenetic analyses. These analyses were executed using four chains for a total of 4 × 10^6^ generations across all datasets, with sampling from Markov chains occurring at intervals of 100 generations. Two independent runs were executed for each analysis. Following the elimination of 30% of samples for the burn-in and assessment of convergence, the remaining samples were retained for in-depth analyses. The resultant topologies were employed to generate 50% majority-rule consensus trees, with posterior probabilities (PP) calculated for relevant clades. The visualization of the phylogenetic trees generated from all analyses was accomplished using FigTree software v.1.4.3 [33].

## 3. Results

### 3.1. Description of Helicotylenchus crassatus Anderson [18] Morphotype 1

**Figure 1:**
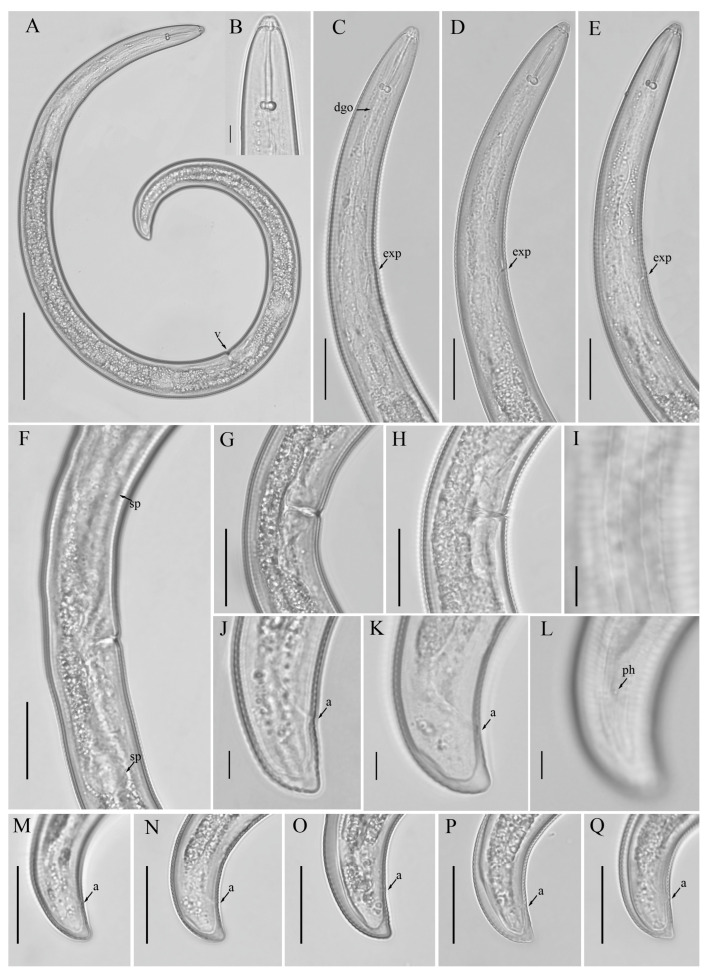
Light micrographs of morphotype 1 *Helicotylenchus crassatus* female Anderson [18]. (**A**) Entire body; (**B**) anterior lip region; (**C**–**E**) pharyngeal regions; (**F**) vulval region with both sets of spermatheca (**G**,**H**) vulval regions; (**I**) lateral field lines; (**J**–**Q**) tail regions. Scale bars: (**A**) 50 μm; (**B**–**H**,**M**–**Q**) 20 μm; (**I**,**J**–**L**) 5 μm. Arrows: (a) anus; (dgo) dorsal pharyngeal gland orifice; (exp) secretory-excretory pore; (sp) spermatheca; (ph) phasmid; (v) vulva.

**Figure 2:**
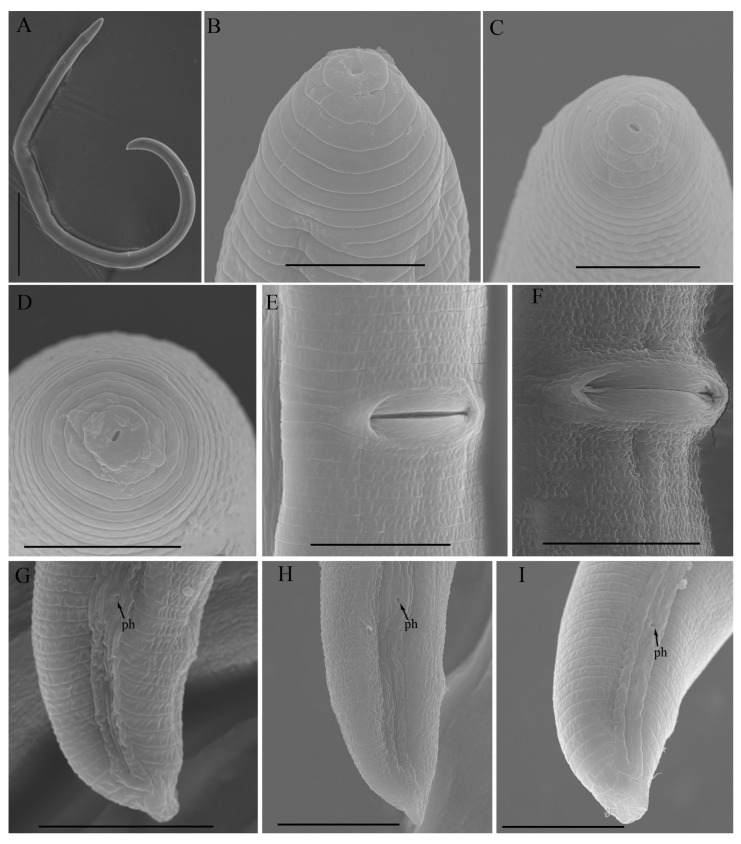
Scanning electron micrographs of morphotype 1 *Helicotylenchus crassatus* female Anderson [18]. (**A**) Entire body; (**B**–**D**) en face view; (**E**,**F**) vulval region; (**G**–**I**) tail regions. Scale bars: (**A**) 100 μm; (**B**–**D**) 5 μm; (**E**–**I**) 10 μm. Arrows: (ph) phasmid.

*Female*: Body assumes a spiral shape after heat relax. Cuticle finely annulated with four incisures in lateral field. Labial disk rounded with an ellipsoidal oral aperture, disk embedded in first lip annulus as observed under scanning electron microscope (SEM), lip region high, continuous with the body, anteriorly flattened, bearing 4–5 annuli. Labial framework strongly sclerotized, with outer margins extending posteriorly 1–2 annuli from basal plate. Stylet robust, with rounded stylet knobs. Orifice of the dorsal pharyngeal gland located less than one half of a stylet length from knobs. Median bulb ovate, with prominent valve plates. Isthmus slender, encircled with nerve ring, pharyngeal gland overlapping intestine ventrally. Secretory-excretory (SE) pore about 4 to 20 annuli anterior to pharyngo-intestinal junction. Hemizonid streak-like immediately or 2–3 annuli anterior to SE pore. Reproductive system didelphic with outstretched ovaries. Spermatheca irregular in shape, almost occupying entire width of pseudocoel. Vulval lip smooth and without any protuberance, invaginated into body, vagina transverse, thick-walled, occupying more than half of corresponding vulval body length. Anus an oblique slit in ventral view, pore-like under SEM. Tail short, bearing 4–9 annuli, conoid in shape, with a prominent dorsal curvature and a somewhat straight ventral side, distal end of terminus without an annulation forming a slight ventral projection. Inner incisures of lateral field terminating in a V or U-shaped pattern on tail, whereas the outer ones form squared terminus as observed under SEM. Phasmids at or three to five annuli anterior to the level of anus (Figure 1 and Figure 2).

*Male*: Not detected.

#### 3.1.1. Description of *Helicotylenchus crassatus* Morphotype 2

**Figure 3:**
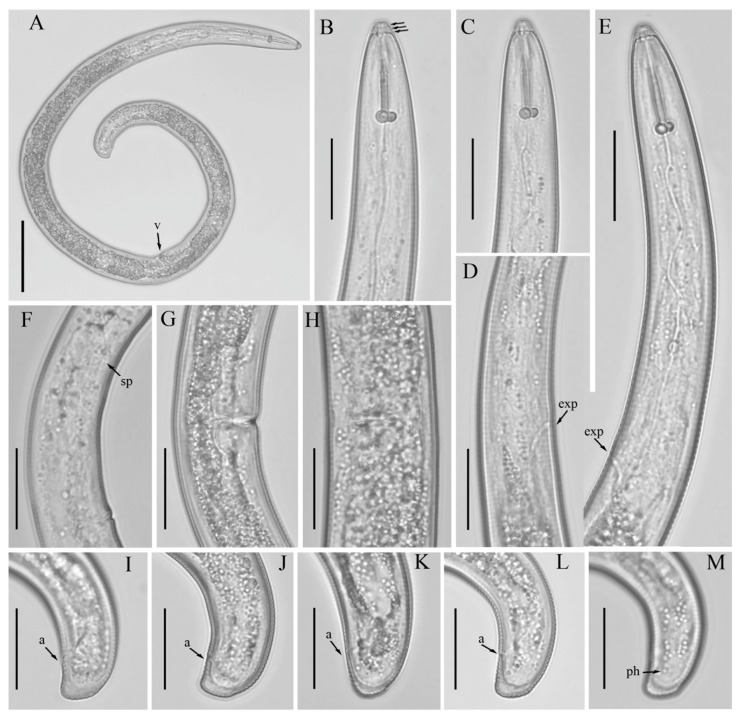
Light micrographs of morphotype 2 *Helicotylenchus crassatus* female Anderson [18], isolate 46. (**A**) Entire body; (**B**,**C**) anterior lip region (arrows in (**B**), indicate the number of annuli on the lip region); (**D**); post pharyngeal region; (**E**) pharyngeal region; (**F**–**H**) vulval region; (**I**–**M**) tail regions. Scale bars: (**A**) 50 μm; (**B**–**M**) 20 μm. Arrows: (a) anus; (exp) secretory-excretory pore; (sp) spermatheca; (ph) phasmid; v (vulva).

**Figure 4:**
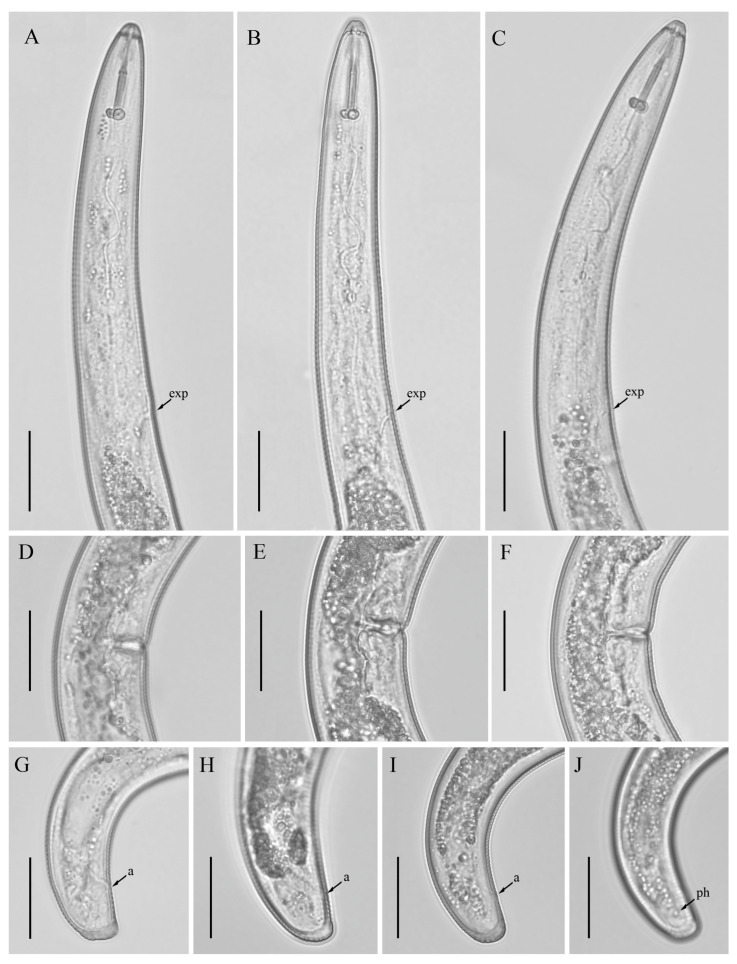
Light micrographs of morphotype 2 *Helicotylenchus crassatus* female Anderson [18], isolates 50, 60, 62. (**A**–**C**) Pharyngeal regions of isolate 50, 60, 62; (**D**–**F**) vulval regions of isolate 50, 60, 62; (**G**–**J**); tail regions of isolate 50, 60, 62. Scale bars: (**A**–**J**) 20 μm. Arrows: (a) anus; (exp) secretory-excretory pore; (ph) phasmid.

**Figure 5:**
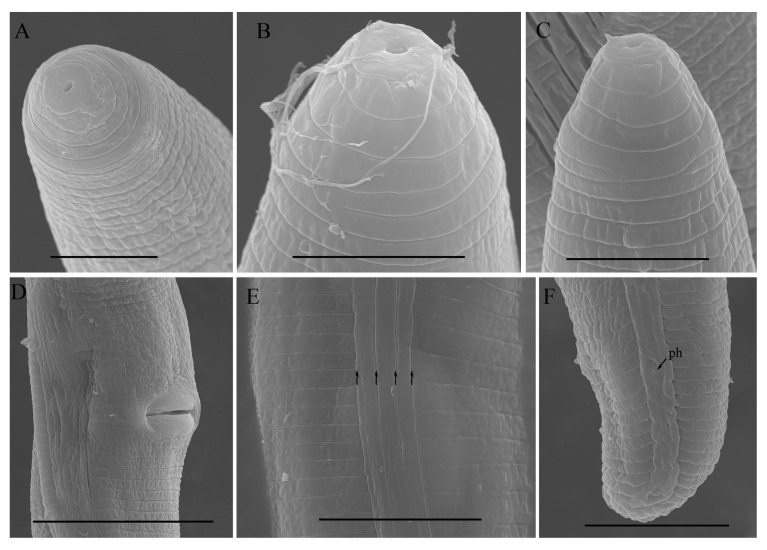
Scanning electron micrographs of morphotype 2 *Helicotylenchus crassatus* female Anderson [18]. (**A**–**C**) En face view; (**D**) post vulval region; (**E**) lateral lines (the arrows indicate the number of lateral lines); (**F**) tail region. Scale bars: (**A**,**C**) 5 μm; (**B**) 4 μm; (**D**) 20 μm; (**E**,**F**) 10 μm. Arrow: (ph) phasmid.

*Female*: Body assumes a close spiral shape after heat relax. Cuticle finely annulated with four incisures in lateral field. Labial disk rounded with ellipsoidal oral aperture, disk embedded in first lip annulus as observed under SEM, lip region high, continuous with body, anteriorly flattened bearing 4–5 annuli. Labial framework sclerotized, with outer margins extending posteriorly 1–2 annuli from basal plate. Stylet robust, with rounded stylet knobs. Orifice of the dorsal pharyngeal gland located less than one-half of stylet length from knobs. Median bulb ovate, with distinct valve plates. Isthmus slender, encircled with a nerve ring, pharyngeal gland overlapping intestine ventrally. Secretory-excretory (SE) pore at or posterior to pharyngo-intestinal junction. Hemizonid 2–3 annuli anterior to SE pore. Reproductive system didelphic with outstretched ovaries. Spermatheca irregular in shape, without sperm. Vulval lip smooth and without any protuberance, invaginated into body, vagina transverse, thick-walled, occupying about half of corresponding vulval body length. Anus an oblique slit in ventral view. Tail annulated, bearing 5–8 annuli, annulation more prominent on dorsal side of tail. Tail shorter in length, dorsally convex-conoid, terminus rounded truncated. Inner incisures of lateral field terminating in a squared shape whereas outer ones form a V-shaped terminus as observed under SEM. Phasmids two to five annuli anterior to level of anus (Figure 3, Figure 4 and Figure 5).

*Male*: Not detected.

#### 3.1.2. Habitat, Locality and Host Associations

The species was initially documented in Ontario, Canada [18] and subsequently reported in several regions of Quebec and Prince Edward Island [14]. After the formal description, the species was only mentioned thrice in the literature, with records from Turkey [34], Morocco [35], and Iran [36], though neither source provided photographic evidence or comprehensive morphometric data. In our study, we identified and characterized both morphotypes of *H. crassatus* in cultivated areas of southern Alberta, with detailed morpho-molecular analyses. Our data matches well with the original description’s characteristics (Table 1). The species was known to be associated with red and white clover, alfalfa, birdsfoot trefoil, pasture grasses, apple, tobacco, alsike clover, dandelion [14], wheat, barley [34] forest soil [35] and walnut [36]. In our case, we encountered both *H. crassatus* morphotypes in fields primarily cultivated with potatoes, integrated into a six-year crop rotation cycle. Moreover, we identified both morphotypes alongside root lesion nematodes and, on occasion, with stunt or pin nematodes. Consequently, the extent of the *H. crassatus* morphotypes feeding on potato plants remains undetermined. A comprehensive investigation is warranted to elucidate the potential threat posed by *H. crassatus* morphotypes, both in isolation and in conjunction with other soil nematode species, to potato crop and subsequent rotational crops.

### 3.2. Designation of Helicotylenchus crassatus Morphotypes

Morphological identification is quite challenging with spiral nematodes, as certain species may exhibit similar or overlapping morphometrical values and share a general morphology. Subbotin et al. [5] designated types A, B, and C for *Helicotylenchus* species that were identified as representatives of the same species but displayed disparities in molecular characteristics. In our study, we designate two morphotypes for *H. crassatus* based on their tail shape, coupled with minor differences in their molecular sequences. Morphotype 1 features a more pronounced conoid tail with a slight ventral projection, while morphotype 2 is characterized by a broadly rounded tail. The 28S and ITS sequences of both *H. crassatus* morphotypes showed 0.8% (6 bp difference) and 2.9% genetic divergence (27 bp difference), respectively, and need to be considered conspecific.

### 3.3. Description of Helicotylenchus oscephalus Anderson [16]

**Figure 6:**
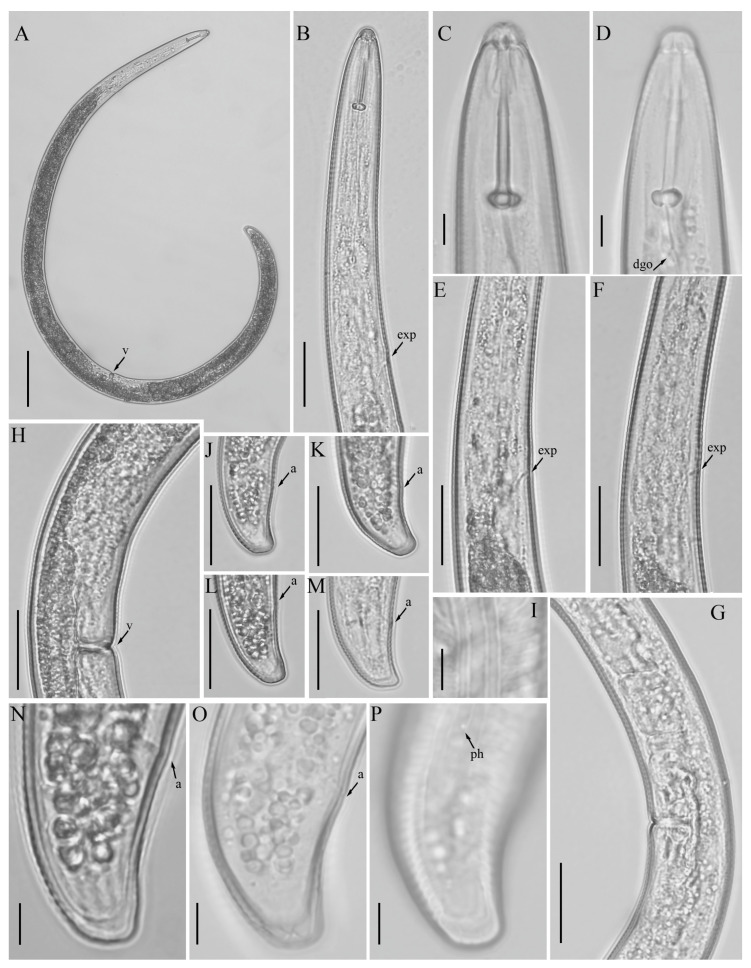
Light micrographs of female *Helicotylenchus oscephalus* Anderson’s [16]. (**A**) Entire body; (**B**) pharyngeal region; (**C**,**D**) anterior lip region; (**E**,**F**) post pharyngeal regions; (**G**,**H**) vulval regions; (**I**) lateral field lines; (**J**–**P**) tail regions. Scale bars: (**A**) 50 μm; (**B**,**E**–**H**,**J**–**M**) 20 μm; (**C**,**D**,**I**,**N**–**P**) 5 μm. Arrows: (a) anus; (dgo) dorsal pharyngeal gland orifice; (exp) secretory-excretory pore; (ph) phasmid; (v) vulva.

**Figure 7:**
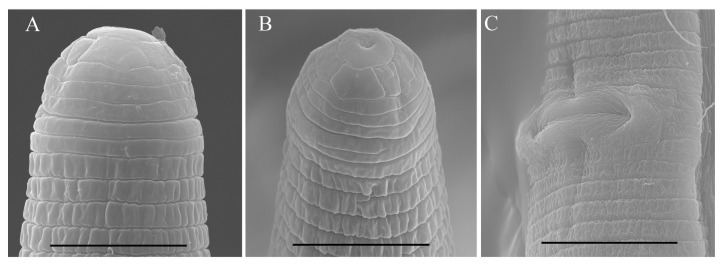
Scanning electron micrographs of female *Helicotylenchus oscephalus* Anderson [16]. (**A**,**B**) En face view; (**C**) vulva region. Scale bars: (**A**,**B**) 5 μm; (**C**) 10 μm.

*Female*: Body assumes an open-to-close spiral shape after heat relax. Cuticle finely annulated with four incisures in lateral field. Labial disk rounded with an ellipsoidal oral aperture, embedded in 6-sectored first annulus as observed under SEM, lip region continuous with body, hemispherical in shape, anteriorly flattened bearing 3–4 annuli. Labial framework strongly sclerotized, with outer margins extending posteriorly 1–2 annuli from basal plate. Stylet robust, with concave stylet knobs. Orifice of dorsal pharyngeal gland located less than one-half of a stylet length from knobs. Median bulb oval, with distinct valve plates. Isthmus slender, encircled with nerve ring, pharyngeal gland overlapping intestine ventrally. Secretory-excretory (SE) pore at or about 5 to 20 annuli posterior to pharyngo-intestinal junction. Hemizonid 2–3 annuli anterior to SE pore. Reproductive system didelphic with outstretched ovaries. Spermatheca irregular in shape, without sperm. Vulval lip smooth and without any protuberance, invaginated into body, vagina transverse, thick-walled, occupying about half of corresponding vulval body length. Anus an oblique slit in ventral view. Tail annulated, bearing 12–19 annuli, with an annulation, more prominent on the dorsal side of tail. Tail moderate in length, dorsally convex-conoid, terminus rounded truncated. Inner incisures of the lateral field terminating in a U-shaped pattern on tail. Phasmids at or two to four annuli anterior to level of anus (Figure 6 and Figure 7).

#### 3.3.1. Habitat, Locality and Host Associations

The species was originally described from British Columbia, Canada, in the rhizosphere of mixed shrub cover [16]. After the initial recognition, the species was only reported from Pakistan in the rhizosphere of *Pinus* sp. [37]. In our study, we found *H. oscephalus* from a dandelion growing on the margin of a potato field. The collected field soil samples were negative for the presence of *H. oscephalus*, suggesting that *H. oscephalus* exhibits a stronger preference for natural vegetation rather than cultivated crops. Our data matches well with the original description’s characteristics, and we did not find any significant variation in morphometrical data of the original and newly detected populations (Table 2).

### 3.4. In Vitro Culture Suitability

**Figure 8:**
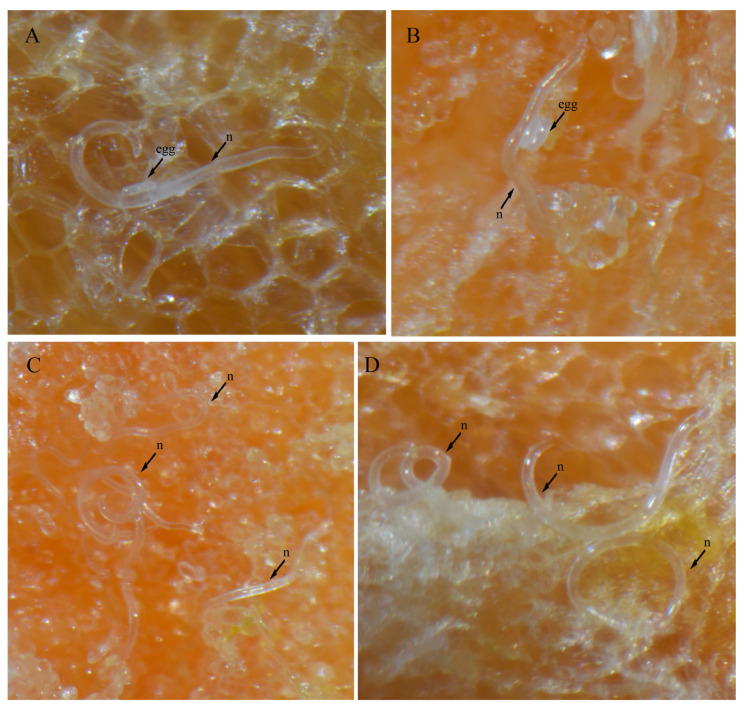
Carrot disk cultures of a morphotype 1 *Helicotylenchus crassatus* Anderson [18]. (**A**) Female with an egg; (**B**) another female with two eggs; (**C**,**D**) several females and juveniles on carrot disks. Microscope magnification: (**A**,**B**,**D**) 90×, (**C**) 67.5×. Arrows: (n) nematode.

**Figure 9:**
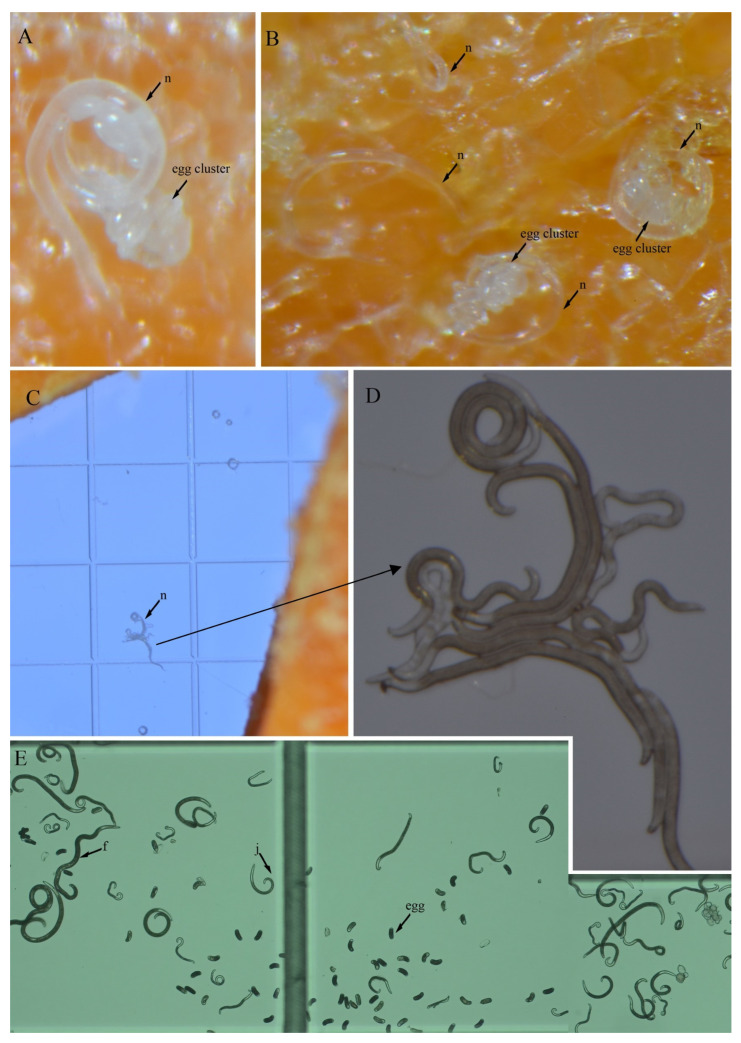
Carrot disk cultures of a morphotype 2 *Helicotylenchus crassatus* Anderson [18]. (**A**) Female with an egg cluster; (**B**) individual females and other females with egg clusters; (**C**,**D**) females that came out of carrot disks; (**E**) females, juveniles and eggs. Microscope magnification: (**A**,**B**,**D**,**E**) 90×, (**C**) 67.5×. Arrows: (f) female, (j) juvenile, (n) nematode.

**Figure 10:**
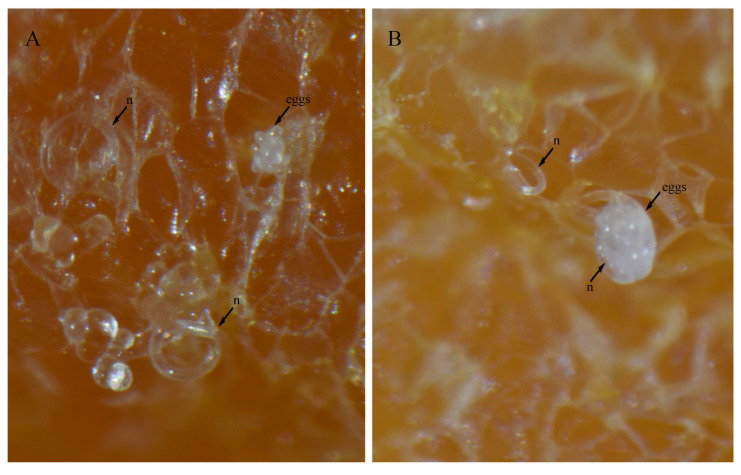
Carrot disk cultures of a *Helicotylenchus oscephalus* Anderson [16]. (**A**,**B**) Female with egg clusters. Microscope magnification: (**A**,**B**) 90×. Arrows: (n) nematode.

To evaluate the feasibility of in vitro culturing for *H. crassatus* morphotypes, and *H. oscephalus*, we introduced 20–25 females onto carrot disks and maintained them at room temperature (20–24 °C) in the dark for a period of three months. A three-month period allows the spiral nematode to reach the population level, which is easy to visualize under a microscope. The carrot disk cultures were prepared as described by Castillo et al. [38]. Our observations revealed the successful multiplication of both morphotypes of *H. crassatus* and *H. oscephalus* on the carrot disks, indicated by the presence of juveniles and several females with eggs (Figure 8, Figure 9 and Figure 10). It is imperative to note that the quantification of reproductive rates was not within the purview of this initial investigation; our primary aim was to test the feasibility of in vitro cultures for *H. crassatus* and *H. oscephalus*.

### 3.5. Phylogenetic Relationships of Detected Species with Other Helicotylenchus Species

**Figure 11:**
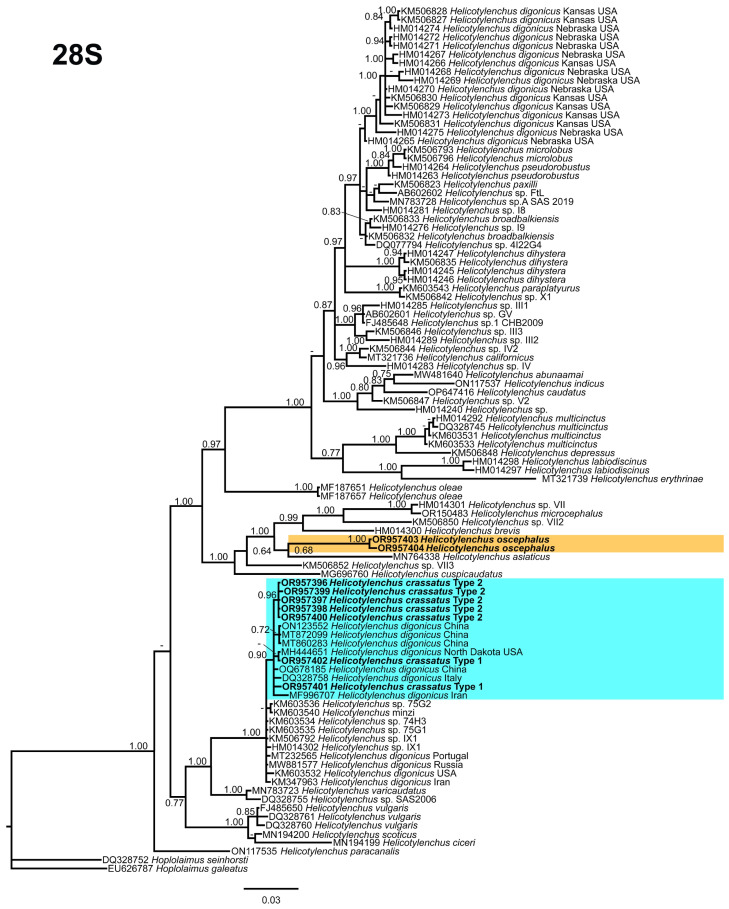
Phylogenetic relationships of the Canadian population of *Helicotylenchus* species with the related species. Bayesian 50% majority rule consensus tree as inferred from D2A–D3B segments of 28S rRNA sequence alignment under the general time-reversible model and a gamma-shaped distribution (GTR + G). Posterior probabilities of greater than 0.70 are provided for the corresponding appropriate clades. The sequences produced in this study are shown in bold, and the colored boxes indicate the clade association of the recovered *Helicotylenchus* species.

In this study, the detected *Helicotylenchus* species were characterized using 18S rRNA, D2–D3 of 28S rRNA, ITS rRNA, and *COI* gene fragments. Phylogenetic trees were constructed for each gene to gain insights into the phylogenetic relationships among the *Helicotylenchus* species. The 28S tree (Figure 11), incorporated 98 sequences of *Helicotylenchus* species retrieved from the NCBI database, with *Hoplolaimus seinhorsti* Luc [39] (DQ328752) and *H. geleatus* (Cobb) Thorne, [40,41] (EU626787) serving as outgroup taxa. Within this tree, both morphotypes of *H. crassatus* formed the basal clade, while *H. oscephalus* occupied a central position, sharing a branch with *H. brevis* (Whitehead) Fortuner [42,43] (HM014300), *H. microcephalus* Sher, [44] (OR150483), and unidentified *Helicotylenchus* species from the USA (HM014301, KM506850). Both morphotypes of *H. crassatus* grouped with *H. digonicus* Perry [45] isolates from diverse regions, including China (ON123552, MT872099, MT860283, OQ678185), the USA (MH444651), Iran (MF996707), and an unidentified *Helicotylenchus* species from Italy (DQ328758). All *H. digonicus* isolates in this grouping lacked essential morphological and morphometrical details, as they had been submitted to the NCBI database without comprehensive documentation. Consequently, their identity as genuine *H. digonicus* or potential misidentified species remains unverified.

**Figure 12:**
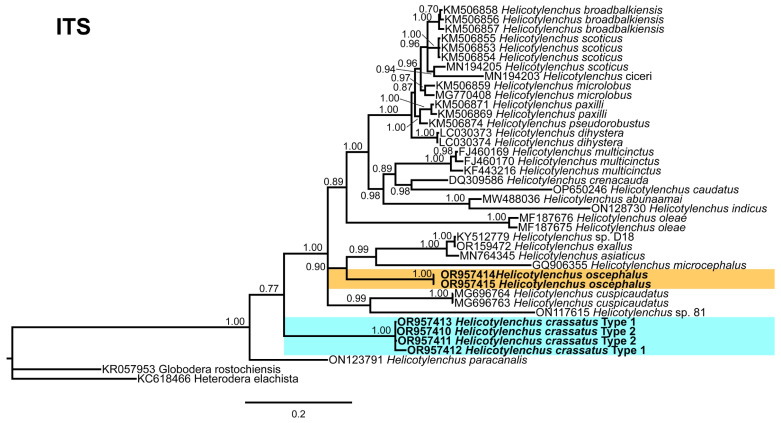
Phylogenetic relationships of the Canadian population of *Helicotylenchus* species with the related species. Bayesian 50% majority rule consensus tree as inferred from ITS rRNA sequence alignment under the general time-reversible model and a gamma-shaped distribution (GTR + G). Posterior probabilities of greater than 0.70 are provided for the corresponding appropriate clades. The sequences produced in this study are shown in bold, and the colored boxes indicate the clade association of the recovered *Helicotylenchus* species.

The ITS tree (Figure 12), comprises 38 sequences of *Helicotylenchus* species, with *Globodera rostochinensis* (Wollenweber) Skarbilovich [46,47] (KR057953) and *Heterodera elachista* Ohshima, [48] (KC618466) as outgroup taxa. Both *H. crassatus* morphotypes were grouped in a distinct branch, while *H. oscephalus* shared a branch with *H. exallus* Sher [44] (OR159472), *H. asiaticus* Mwamula, Na, Kim, Kim, Han, & Lee [13] (MN764345), *H. microcephalus* (GQ906355), and an unidentified *Helicotylenchus* species from Korea (KY512779).

**Figure 13:**
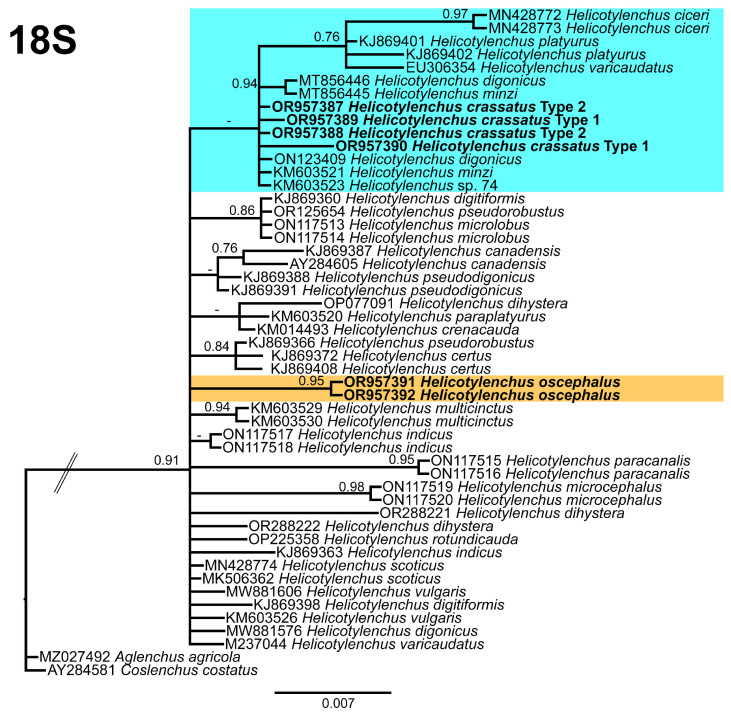
Phylogenetic relationships of the Canadian population of *Helicotylenchus* species with the related species. Bayesian 50% majority rule consensus tree as inferred from 18S rRNA sequence alignment under the general time-reversible model with invariable sites and a gamma-shaped distribution (GTR + I + G). Posterior probabilities of greater than 0.70 are provided for the corresponding appropriate clades. The sequences produced in this study are shown in bold, and the colored boxes indicate the clade association of the recovered *Helicotylenchus* species.

The 18S tree (Figure 13), was constructed with 50 sequences of *Helicotylenchus* species, with *Aglenchus agricola* (de Man) Andrássy [49,50] (MZ027492) and *Coslenchus costatus* (de Man) Siddiqi [51,52] (AY284581) as outgroup taxa. This tree is not well resolved; the *Helicotylenchus* species do not form distinct clades. Both *H. crassatus* morphotypes were grouped in the first clade of the tree as separate branches, whereas *H. oscephalus* grouped independently in the middle of the tree.

**Figure 14:**
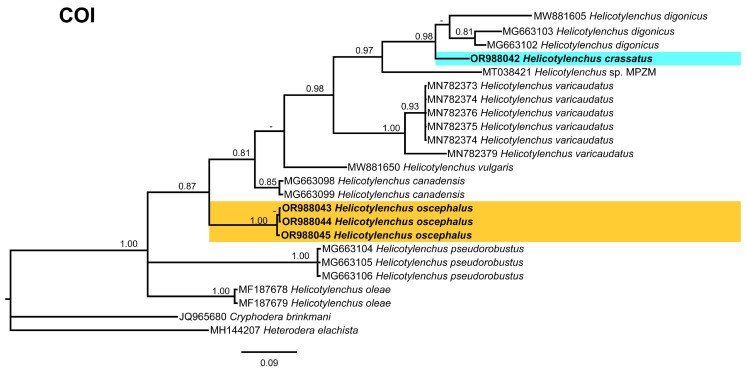
Phylogenetic relationships of the Canadian population of *Helicotylenchus* species with the related species. Bayesian 50% majority rule consensus tree as inferred from *COI* gene of mitochondrial DNA sequence alignment under the general time-reversible model with invariable sites and a gamma-shaped distribution (GTR + G + I). Posterior probabilities of greater than 0.70 are provided for the corresponding appropriate clades. The sequences produced in this study are shown in bold, and the colored boxes indicate the clade association of the recovered *Helicotylenchus* species.

Considering the limited *COI* sequence data for *Helicotylenchus* species, the *COI* tree (Figure 14), included 23 sequences of *Helicotylenchus* species from the NCBI, including Cryphodera brinkmani Karssen & Aelst [53] (JQ965680) and *Heterodera elachista* (MH144207) as outgroup taxa. Only morphotype 1 was included in this analysis, and its sequence clustered with *H. digonicus* from Russia (MW881605) and Poland (MG663103, MG663102), while *H. oscephalus* formed an independent branch in the middle of the tree. The scarcity of *COI* data emphasizes the need for further exploration and the comprehensive assessment of morphological and molecular characteristics, particularly for isolates with potential misidentifications, to enhance the accuracy of nematode species identification.

## 4. Discussion

The genus *Helicotylenchus* is known for its remarkable morphological diversity and various parasitic strategies [5,6]. Some act as ectoparasites, feeding on the root surface, while others display semi-endoparasitic behavior by embedding themselves 4–6 cells deep within the cortex. For instance, *H. multicinctus* adopts a fully endoparasitic lifestyle, entering the roots to feed and lay eggs [12]. Infestation by these nematodes typically leads to symptoms such as stunting, sparse yellow foliage, root necrosis, and dieback [12]. Given the *H. crassatus* morphotypes, and the *H. oscephalus* identified in this study were isolated from soil samples, we anticipate that these species exhibit ectoparasitic feeding behavior. It was also observed in our carrot disk cultures that females only insert their anterior region for feeding. The literature indicates that the genus displays two primary modes of reproduction: parthenogenetic, involving asexual reproduction where females give rise to female offspring, and amphimixis, necessitating both male and female for reproduction. No males were found for any of our detected species. We presume, under Alberta’s climatic and edaphic conditions, that the *H. crassatus* morphotypes and *H. oscephalus* multiply through parthenogenesis.

The amphimixis mode of reproduction is challenging because it is likely that nematodes would not mate within an artificial environment. The parthenogenic reproductive strategy prompted us to initiate in vitro cultures of the detected species. A similar approach was used by Xia et al. [54] in China, where the authors transferred females of *H. microlobus* Perry [45] onto carrot disks and were successful in obtaining a mass culture of *H. microlobus.* Our study, representing the second of its kind, substantiates that certain spiral nematode species can indeed be cultured artificially.

In addition to spiral nematodes, the root lesion nematode is reported to be the most dominating and prevalent nematode in southern Alberta’s soils [19], and it is mostly co-detected with spiral nematodes. The collective impact of spiral and root lesion nematodes on Alberta’s cropping system remains unclear. This uncertainty could be linked to the practice of composite soil sampling [55]. This practice provides homogeneity in the nematode count, but may introduce a degree of variability affecting the precise distribution and impact of *Helicotylenchus* species on cultivated crops. Given the strong emphasis on sustainable agriculture in Alberta’s farming community, the availability of in vitro cultures for spiral nematodes holds the potential to furnish valuable threshold and pathogenicity assessments. Moreover, this approach can allow us to ascertain whether spiral nematodes inflict equivalent or potentially greater harm compared to root lesion nematodes.

The phylogenetic analysis of the identified populations indicated close relationships between both morphotypes of *H. crassatus* and certain isolates of *H. digonicus*. It is observed that some isolates of *H. digonicus* cluster within different clades of the phylogenetic tree. The widespread distribution and diverse host range of *H. digonicus* raise concerns regarding the accuracy of identifications, suggesting the possibility of misidentification in some instances or the presence of a species complex within the *H. digonicus* morphospecies. Hence, there is a pressing need for a comprehensive reassessment that encompasses both morphological and molecular characteristics to ensure the accurate classification of these *H. digonicus* isolates. Notably, *H. oscephalus* exhibited an independent grouping in all analyzed trees, highlighting its unique position within the phylogenetic framework. In conclusion, the molecular characterization presented herein provides a comprehensive set of sequences that can serve as a valuable resource for future genetic studies.

Soil is an integral component of the biosphere and crucial for agriculture. It houses various soil nematodes, many of which are associated with plants without causing any harm [56,57]. However, it is vital to recognize that in agricultural systems, the collective impact of certain nematode species can intensify over time, resulting in a diminished crop quality and yield. In our current investigation, we identified two *Helicotylenchus* species, none of the recovered species presently qualify as pest species. Nevertheless, given the rapidly changing climatic and biotic conditions, these species could evolve into genuine pathogens. Considering the prohibition and restriction of many nematicides, it is prudent to prioritize the early and precise detection of nematode populations in agricultural lands. Additionally, research efforts should concentrate on assessing their impact on crop rotations to formulate effective management strategies.

## Figures and Tables

**Table 1:** Comparative morphometrics of both female *Helicotylenchus crassatus* Anderson [18] reported in this study and in the original description. This study’s measurements are in μm and in the form of mean ± standard deviation (range), whereas the original description measurements are in μm and in the form of means (ranges).

	This Study					Anderson [18]
Location	Southern Alberta, Canada					Ontario, Canada
Characteristics	Morphotype 1	Morphotype 2				
	Population 93	Population 50	Population 46	Population 60	Population 61	Original Population
n	17	17	10	10	10	20
Body length (L)	647.0 ± 42.2 (584.0–747.0)	768.0 ± 66.0 (659.0–866.0)	798 ± 51.2 (733.0–900.0)	799.0 ± 45.8 (720.0–851.0)	792.0 ± 75.7 (672.0–891.0)	720 (646–779)
a	26.0 ± 2.1 (21.0–30.0)	31.0 ± 2.6 (28.0–37.0)	29.0 ± 2.3 (25.0–32.0)	29.0 ± 2.7 (26.0–34.0)	27.5 ± 2.6 (22.0–31.0)	31 (27–34)
b	5.5 ± 0.5 (5.0–7.0)	5.9 ± 0.5 (4.7–6.6)	6.0 ± 0.4 (5.5–7.0)	6.3 ± 0.3 (6.0–7.0)	6.3 ± 0.6 (5.0–7.0)	5.7 (5.2–6.2)
b’	4.7 ± 0.5 (4.0–6.0)	5.0 ± 0.4 (4.0–5.5)	5.4 ± 0.3 (5.0–6.0)	5.0 ± 0.3 (5.0–6.0)	5.0 ± 0.5 (4.2–6.0)	4.7 (4.3–5.0)
c	53.0 ± 7.0 (45.0–67.0)	61.0 ± 4.5 (53.0–70.0)	64.0 ± 4.5 (57.0–70.0)	61.5 ± 6.6 (53.0–72.0)	62.0 ± 9.8 (48.0–81.0)	59 (45–71)
c’	1.1 ± 0.1 (0.8–1.4)	0.9 ± 0.1 (0.7–1.1)	0.8 ± 0.1 (0.7–1.0)	0.8 ± 0.1 (0.8–1.0)	0.8 ± 0.1 (0.6–1.0)	0.79 (0.63–1.00)
O	30.0 ± 3.6 (23.0–36.0)	32.0 ± 2.9 (27.5–38.0)	34.5 ± 3.5 (31.0–42.0)	30.7 ± 2.0 (28.0–35.0)	32.0 ± 3.4 (27.0–37.5)	28 (20–36)
V	62.0 ± 2.3 (57.0–67.0)	61.0 ± 1.6 (57.0–63.0)	63.0 ± 2.3 (59.0–66.0)	62.0 ± 1.6 (58.5–64.0)	62.0 ± 1.1 (60.0–63.0)	–
Lip region height	3.5 ± 0.4 (3.0–4.0)	3.5 ± 0.2 (3.0–3.8)	3.4 ± 0.3 (3.0–3.8)	3.7 ± 0.4 (3.0–4.2)	3.7 ± 0.4 (3.2–4.3)	–
Lip region width	7.0 ± 0.5 (6.0–8.0)	6.8 ± 0.4 (6.0–7.5)	6.8 ± 0.5 (6.0–7.4)	6.9 ± 0.3 (6.3–7.4)	6.9 ± 0.5 (6.0–7.3)	–
Stylet length	26.0 ± 1.2 (24.0–28.0)	27.0 ± 1.0 (25.0–28.0)	26.0 ± 1.4 (24.0–28.0)	27.0 ± 1.0 (25.0–28.0)	27.0 ± 1.3 (24.5–28.0)	28 (26–30)
Stylet knob height	3.0 ± 0.4 (2.0–3.4)	2.5 ± 0.2 (2.0–3.0)	3.0 ± 0.3 (2.4–3.3)	3.4 ± 0.4 (3.0–3.9)	2.9 ± 0.3 (2.5–3.3)	–
Stylet knob width	5.0 ± 0.3 (4.3–5.5)	5.0 ± 0.4 (4.5–6.0)	5.5 ± 0.2 (5.2–5.5)	5.4 ± 0.3 (5.0–6.0)	5.3 ± 0.3 (5.0–6.0)	–
DGO distance from stylet knob base	8.0 ± 0.9 (6.5–9.0)	8.8 ± 0.7 (7.5–10.0)	8.9 ± 1.0 (8.0–10.5)	8.0 ± 0.6 (7.5–9.5)	8.8 ± 0.7 (7.5–10.0)	–
Median bulb length	12.3 ± 1.0 (10.0–14.0)	12.5 ± 1.0 (10.5–14.0)	13.5 ± 0.9 (12.5–15.5)	13.5 ± 1.1 (12.5–15.0)	14.0 ± 0.8 (12.0–15.0)	–
Median bulb width	10.0 ± 0.8 (8.0–11.5)	10.0 ± 0.9 (8.0–11.0)	11.0 ± 1.0 (9.0–12.5)	11.0 ± 1.2 (9.0–13.0)	11.0 ± 0.8 (10.0–12.5)	–
Anterior end to excretory pore	101.0 ± 3.3 (91.0–106.0)	118.5 ± 5.6 (112.0–129.0)	118.0 ± 6.3 (110.0–128.0)	119.0 ± 5.7 (111.0–129.0)	120.0 ± 5.0 (111.0–131.0)	(106–125)
Pharynx length (till pharyngo-intestinal junction)	119.0 ± 7.7 (95.0–128.0)	130.0 ± 6.0 (121.0–147.0)	126.5 ± 7.4 (118.5–140.0)	126.0 ± 5.8 (117.0–136.0)	127.0 ± 5.7 (115.5–133.0)	(114–134)
Pharynx length (till pharyngeal lobe)	139.0 ± 9.0 (111.0–149.0)	157.0 ± 10.4 (140.0–175.5)	148.0 ± 7.2 (139.0–163.0)	154.0 ± 6.7 (145.5–169.0)	158.0 ± 8.9 (143.0–176.0)	(141–163)
Maximum body diam.	25.0 ± 2.1 (20.0–29.0)	24.5 ± 2.5 (21.0–30.7)	27.5 ± 2.2 (24.5–32.0)	27.0 ± 2.8 (22.0–31.0)	29.0 ± 2.0 (25.0–31.0)	–
Vulva body diam.	23.0 ± 2.1 (19.0–28.0)	24.0 ± 2.6 (20.0–29.0)	25.5 ± 2.3 (22.0–29.0)	26.5 ± 1.7 (24.5–29.0)	26.0 ± 1.7 (23.0–28.0)	–
Distance from vulva to tail terminus	248.0 ± 28.2 (192.0–320.0)	470.0 ± 39.5 (412.0–543.0)	298.5 ± 32.0 (258.0–358.0)	306.0 ± 24.3 (272.0–338.0)	301.5 ± 28.8 (248.0–336.0)	–
Anal body diam.	13.6 ± 1.0 (11.5–15.3)	15.0 ± 1.6 (12.0–18.0)	16.0 ± 1.8 (12.0–18.0)	15.5 ± 1.5 (13.0–17.5)	16.0 ± 1.9 (14.0–20.0)	–
Tail length	12.3 ± 1.6 (10.0–15.0)	12.6 ± 1.1 (11.0–15.0)	12.5 ± 0.9 (11.0–14.0)	13.0 ± 1.7 (10.0–15.0)	12.5 ± 1.4 (11.0–15.0)	12 (10–17)
Hyaline tail region	3.9 ± 0.5 (3.0–4.6)	2.7 ± 0.5 (2.3–4.0)	3.6 ± 0.7 (2.6–4.5)	3.8 ± 0.6 (3.0–4.9)	4.0 ± 0.7 (3.0–4.6)	–

Abbreviations: n, number of specimens on which the measurements are based; a, body length/greatest body diameter; b, body length/distance from the anterior end to the pharyngo-intestinal junction; b’ body length/distance from the anterior end to the posterior end of the pharyngeal glands; c, body length/tail length; c’, tail length/tail diameter at the anus; O, distance of the dorsal pharyngeal gland opening posterior to the stylet knobs expressed as a percentage of the stylet length; V, distance from the body’s anterior end to the vulva as a percentage (%) of the body length; DGO, dorsal pharyngeal gland orifice. ‘–’ data are not available.

**Table 2:** The comparative morphometrics of female *Helicotylenchus oscephalus* Anderson [16] reported in this study and in the original description. This study’s measurements are in μm and in the form of mean ± standard deviation (range) whereas the original description measurements are in μm and in the form of means (ranges).

Characteristics	This Study	Anderson [16]
Population	Southern Alberta, Canada	British Columbia, Canada
n	17	12
Body length (L)	876.6 ± 45.9 (802.0–963.0)	857 (782–927)
a	29.5 ± 1.5 (27.3–32.2)	32 (28–35)
b	6.5 ± 0.3 (6.0–7.0)	6.2 (5.7–6.6)
b’	5.2 ± 0.2 (4.9–5.7)	5.1 (4.6–5.5)
c	37.4 ± 2.8 (31.2–41.5)	33 (27–39)
c’	1.3 ± 0.1 (1.2–1.5)	1.3 (1.1–1.7)
O	27.8 ± 1.2 (26.5–30.0)	28 (21–33)
V	62.1 ± 1.4 (58.8–64.5)	61 (58–63)
Lip region height	35 ± 0.3 (3.0–4.0)	–
Lip region width	7.4 ± 0.4 (7.0–8.3)	–
Stylet length	29.5 ± 0.9 (28.0–31.0)	27 (25–28)
Stylet knob height	2.7 ± 0.5 (2.1–4.0)	–
Stylet knob width	5.1 ± 0.3 (4.6–5.5)	–
DGO distance from stylet knob base	8.3 ± 0.3 (8.0–9.0)	(6–9)
Median bulb length	14.5 ± 0.7 (12.5–15.4)	–
Median bulb width	11.3 ± 0.8 (10.0–12.4)	–
Anterior end to excretory pore	137.5 ± 4.1 (130.0–144.0)	135 (127–143)
Pharynx length (till pharyngo-intestinal junction)	134.8 ± 4.4 (123.0–139.0)	139 (128–153)
Pharynx length (till pharyngeal lobe)	167.9 ± 8.6 (156.0–180.0)	169 (156–185)
Maximum body diam.	29.8 ± 2.1 (25.0–33.0)	(25–28)
Vulva body diam.	27.4 ± 2.4 (24.0–31.0)	–
Distance from vulva to tail terminus	331.0 ± 25.1 (284.0–371.0)	–
Anal body diam.	17.8 ± 1.6 (15.0–20.0)	–
Tail length	23.6 ± 2.3 (20.0–28.0)	26 (21–29)
Phasmid position from tail terminus	27.6 ± 1.3 (27.0–30.0)	–

Abbreviations: n, number of specimens on which the measurements are based; a, body length/greatest body diameter; b, body length/distance from the anterior end to the pharyngo-intestinal junction; b’ body length/distance from the anterior end to the posterior end of pharyngeal glands; c, body length/tail length; c’, tail length/tail diameter at the anus; O, distance of the dorsal pharyngeal gland opening posterior to the stylet knobs expressed as a percentage of the stylet length; V, distance from the body’s anterior end to the vulva as a percentage (%) of the body length; DGO, dorsal pharyngeal gland orifice. ‘–’ data are not available.

## Data Availability

Data are contained within the article.

## References

[1] Steiner G. (1945). *Helicotylenchus*, a new genus of plant-parasitic nematodes and its relationship to *Rotylenchus* Filipjev. Proc. Helminthol. Soc. Wash..

[2] Siddiqi M.R. (2000). Tylenchida: Parasites of Plants and Insects.

[3] Inserra R., Vovlas N., Golden A.M. (1979). *Helicotylenchus oleae* n. sp. and *H. neopaxilli* n. sp.(Hoplolaimidae), two new spiral nematodes parasitic on olive trees in Italy. J. Nematol..

[4] Karakaş M. (2007). Life cycle and mating behavior of *Helicotylenchus multicinctus* (Nematoda: Hoplolaimidae) on excised *Musa cavendishii* roots. Biologia.

[5] Subbotin S.A., Inserra R.N., Marais M., Mullin P., Powers T.O., Roberts P.A., Van Den Berg E., Yeates G.W., Baldwin J.G. (2011). Diversity and phylogenetic relationships within the spiral nematodes of *Helicotylenchus* Steiner, 1945 (Tylenchida: Hoplolaimidae) as inferred from analysis of the D2-D3 expansion segments of 28S rRNA gene sequences. Nematology.

[6] Subbotin S.A., Vovlas N., Yeates G.W., Hallmann J., Kiewnick S., Chizhov V.N., Manzanilla-López R.H., Inserra R.N., Castillo P. (2015). Morphological and molecular characterisation of *Helicotylenchus pseudorobustus* (Steiner, 1914) Golden, 1956 and related species (Tylenchida: Hoplolaimidae) with a phylogeny of the genus. Nematology.

[7] Cobb N.A. (1893). Nematodes, Mostly Australian and Fijian.

[8] Golden A.M. (1956). Taxonomy of the spiral nematodes (*Rotylenchus* and *Helicotylenchus*), and the developmental stages and host-parasite relationships of *R. buxophilus*, n. sp., attacking boxwood. Bull. Md. Agric. Exp. Stn..

[9] Steiner G. (1914). Freilebende nematoden aus der Schweiz. 2. Teil. einer vorlafifigen Mitteilung. Arch. Hydrobiol. Planktork..

[10] Yuen P.H. (1964). Four new species of *Helicotylenchus* Steiner (Hoplolaiminae: Tylenchida) and a redescription of *H. canadensis* Waseem, 1961. Nematologica.

[11] McSorley R., Parrado J. (1986). Nematological reviews: *Helicotylenchus multicinctus* on bananas: An international problem. Nematropica.

[12] O’Bannon J., Inserra R. (1989). Helicotylenchus Species as Crop Damaging Parasitic Nematodes. Nematology Circular 165.

[13] Mwamula A.O., Na H., Kim Y.H., Kim Y.H., Han G., Lee D.W. (2020). Characterization of a new spiral nematode, *Helicotylenchus asiaticus* n. sp. and three known species from Korea; with comments on the validity of *Helicotylenchus microlobus* Perry in Perry, Darling & Thorne, 1959. Eur. J. Plant Pathol..

[14] Anderson R. (1974). Canadian species of the genus *Helicotylenchus* Steiner, 1945 (Nematoda: Hoplolaimidae), their identifying characteristics and descriptions of three new species. Can. J. Zool..

[15] Anderson R. (1978). *Helicotylenchus urobelus* n. sp.(Nematoda: Hoplolaimidae) from New Brunswick, Canada. Can. J. Zool..

[16] Anderson R. (1979). A supplemental key to species of *Helicotylenchus* Steiner, 1945 (Nematoda: Hoplolaimidae) described since 1972 and a description of *H. oscephalus* n. sp. Can. J. Zool..

[17] Anderson R., Eveleigh E. (1982). Description of *Helicotylenchus amplius* n. sp. and a key to the Canadian species of the genus (Nematoda: Hoplolaimidae). Can. J. Zool..

[18] Anderson R. (1973). Morphology and description of *Helicotylenchus crassatus* n. sp. (Nematoda: Hoplolaimidae) from eastern Canada. Can. J. Zool..

[19] Forge T.A., Larney F.J., Kawchuk L.M., Pearson D.C., Koch C., Blackshaw R.E. (2015). Crop rotation effects on *Pratylenchus neglectus* populations in the root zone of irrigated potatoes in southern Alberta. Can. J. Plant Pathol..

[20] Jenkins W. (1964). A rapid centrifugal-flotation technique for separating nematodes from soil. Plant Dis. Rep..

[21] Seinhorst J. (1959). A rapid method for the transfer of nematodes from fixative to anhydrous glycerin. Nematologica.

[22] Grisse D. (1969). Redescription ou modification de quelques techniques utilisées dans l’étude des nématodes phytoparasitaires. Meded. Van De Rijks Fac. Landbouwwet. Gent.

[23] Maria M., Powers T., Tian Z., Zheng J. (2018). Distribution and description of criconematids from Hangzhou, Zhejiang Province. China J. Nematol..

[24] Holterman M., van der Wurff A., van den Elsen S., van Megen H., Bongers T., Holovachov O., Bakker J., Helder J. (2006). Phylum-wide analysis of SSU rDNA reveals deep phylogenetic relationships among nematodes and accelerated evolution toward crown clades. Mol. Biol. Evol..

[25] De Ley P., Felix M.-A., Frisse L., Nadler S., Sternberg P., Thomas W.K. (1999). Molecular and morphological characterisation of two reproductively isolated species with mirror-image anatomy (Nematoda: Cephalobidae). Nematology.

[26] Vrain T., Lalik B. (1983). Distribution and pathogenicity of the alfalfa stem nematode, *Ditylenchus dipsaci,* in British Columbia. Plant Dis..

[27] Bowles J., Blair D., McManus D.P. (1992). Genetic variants within the genus *Echinococcus* identified by mitochondrial DNA sequencing. Mol. Biochem. Parasitol..

[28] Derycke S., Remerie T., Vierstraete A., Backeljau T., Vanfleteren J., Vincx M., Moens T. (2005). Mitochondrial DNA variation and cryptic speciation within the free-living marine nematode *Pellioditis marina*. Mar. Ecol. Prog. Ser..

[29] Katoh K., Rozewicki J., Yamada K.D. (2019). MAFFT online service: Multiple sequence alignment, interactive sequence choice and visualization. Brief. Bioinform..

[30] Hall T.A. (1999). BioEdit: A User-Friendly Biological Sequence Alignment Editor and Analysis Program for Windows 95/98/NT. Nucleic Acids Symp. Ser..

[31] Tan G., Muffato M., Ledergerber C., Herrero J., Goldman N., Gil M., Dessimoz C. (2015). Current methods for automated filtering of multiple sequence alignments frequently worsen single-gene phylogenetic inference. Syst. Biol..

[32] Darriba D., Taboada G., Doallo R., Posada D. (2012). jModelTest 2: More models, new heuristics and parallel computing. Nat. Meth..

[33] Rambaut A. FigTree. http://tree.bio.ed.ac.uk/software/figtree.

[34] Uludamar E.B.K., Yildiz Ş., Imren M., Atilla Ö., ElekçİoĞlu İ.H. (2018). Occurrence of plant parasitic nematode species in important crops in the Southeast Anatolia Region of Turkey. Turk. J. Entomol..

[35] Mateille T., Tavoillot J., Martiny B., Dmowska E., Winiszewska G., Ferji Z., Msanda F., El Mousadik A. (2016). Aridity or low temperatures: What affects the diversity of plant-parasitic nematode communities in the Moroccan argan relic forest. Appl. Soil Ecol..

[36] Bahmani J., Khozeini F., Barooti S., Rezaee S., Ghaderi R. (2013). Plant-parasitic nematodes associated with walnut in the Sanandej region of west Iran. J. Plant Prot. Res..

[37] Ishaque U., Kazi N. (2022). Some new records of *Helicotylenchus* (steiner, 1945) species from pakistan (nematoda: Hoplolaimidae). Plant Prot..

[38] Castillo P., Trapero-Casas J., Jiménez-Díaz R. (1995). Effect of time, temperature, and inoculum density on reproduction of *Pratylenchus thornei* in carrot disk cultures. J. Nematol..

[39] Luc M. (1958). Nematodes and wilting in cotton in Southwestern Madagascar. Coton Et Fibres Trop..

[40] Cobb N. (1913). New nematode genera found inhabiting fresh water and non-brackish soils. J. Wash. Acad. Sci..

[41] Thorne G. (1935). Notes on free-living and plant-parasitic nematodes, II. Proc. Helminthol. Soc. Wash..

[42] Whitehead A. (1958). *Rotylenchoides brevis* ng, n. sp. (Rotylenchoidinae n. subfam.: Tylenchida). Nematologica.

[43] Fortuner R. (1984). Morphometrical variability in *Helicotylenchus* Steiner, 1945. 6: Value of the characters used for specific identification. Rev. De Nématologie.

[44] Sher S. (1966). Revision of the hoplolaiminae (nematoda) VI. *Helicotylenchus* steiner, 1945 1. Nematologica.

[45] Perry V.G., Darling H.M., Thorne G. (1959). Anatomy, taxonomy and control of certain spiral nematodes attacking blue grass in Wisconsin. Res. Bull..

[46] Wollenweber H. (1923). Krankheiten und Beschädigungen der Kartoffel. Arbeiten des Forschungsinstitutes für Kartoffelbau, Heft 7.

[47] Skarbilovich T. (1959). On the structure of systematics of nematodes order Tylenchida Thorne, 1949. Acta Parasitol. Pol..

[48] Ohshima Y. (1974). *Heterodera elachista* n. sp., an upland rice cyst nematode from Japan. Jpn J. Nematol..

[49] de Man J.G. (1884). Die, Frei in der Reinen Erde und im Süssen Wasser Lebenden Nematoden der Niederländischen Fauna: Eine Systematisch-faunistische Monographie.

[50] Andrássy I. (1954). Revision der Gattung *Tylenchus* Bastian, 1865 (Tylenchidae, Nematoda). Acta Zool. Hung.

[51] de Man J.G. (1921). Nouvelles recherches sur les nématodes libres terricoles de la Hollande. Capita Zool..

[52] Siddiqi M.R. (1978). The unusual position of the phasmids in *Coslenchus costatus* (de Man, 1921) gen. n., comb. n. and other Tylenchidae (Nematoda: Tylenchida). Nematologica.

[53] Karssen G., Van Aelst A. (1999). Description of *Cryphodera brinkmani* n. sp.(Nematoda: Heteroderidae), a parasite of *Pinus thunbergii* Parlatore from Japan, including a key to the species of the genus *Cryphodera* Colbran, 1966. Nematology.

[54] Xia Y.-H., Li J., Xu F.-F., Lei B., Li H.-L., Wang K., Li Y. (2022). Identification and a culture method for a *Helicotylenchus microlobus* from tomato in China. BMC Zool..

[55] Abd-Elgawad M.M. (2021). Optimizing sampling and extraction methods for plant-parasitic and entomopathogenic nematodes. Plants.

[56] Munawar M., Yevtushenko D.P., Castillo P. (2021). Overview of the genus *Boleodorus* and first reports of *Boleodorus thylactus* and *B. volutus* from southern Alberta, Canada. Animals.

[57] Munawar M., Castillo P., Yevtushenko D.P. (2022). Description of *Filenchus* species from agroecosystem of southern Alberta, Canada. Agronomy.

